# Systematic pan-cancer analysis identifies gasdermin B as an immunological and prognostic biomarker for kidney renal clear cell carcinoma

**DOI:** 10.3389/fonc.2023.1164214

**Published:** 2023-03-30

**Authors:** Xuehe Liu, Feiyan Xie, Jin Ding, Suhua Li, Jixi Li

**Affiliations:** ^1^ State Key Laboratory of Genetic Engineering, School of Life Sciences and Huashan Hospital, Shanghai Engineering Research Center of Industrial Microorganisms, MOE Engineering Research Center of Gene Technology, Fudan University, Shanghai, China; ^2^ Clinical Cancer Institute, Center for Translational Medicine, Naval Medical University, Shanghai, China; ^3^ Division of Natural Science, Duke Kunshan University, Jiangsu, China

**Keywords:** gasdermin B, pan-cancer analysis, KIRC, prognostic, immune infiltrate, TNFRSF25, TNFSF14

## Abstract

Gasdermin (GSDM)-mediated cell lytic death plays an essential role in immunity and tumorigenesis. Despite the association of gasdermin B (GSDMB) with the tumorigenesis of various cancers, whether GSDMB functions as a prognostic biomarker in renal cell carcinoma remains poorly understood. Here, we explored the potential immunological functions and the prognostic value of GSDMB across multiple tumors with The Cancer Genome Atlas (TCGA) and Genotype-Tissue Expression (GTEx) databases, including analyzing the relationship between GSDMB expression and prognosis, tumor–immune system interactions, immunomodulators, and immune cell infiltration of different tumors. Importantly, elevated expression of GSDMB is an essential factor for the poor prognosis of kidney renal clear cell carcinoma (KIRC) patients, suggesting that it might be helpful to predict a survival benefit from a clinical therapy regimen. Furthermore, GSDMB expression promoted the level of CD4+ T-cell infiltration of the tumors but is significantly negatively associated with immature dendritic cells (iDCs) in KIRC. Additionally, we identified TNFRSF25 and TNFSF14 as immunostimulators highly correlated with GSDMB expression. Kyoto Encyclopedia of Genes and Genomes (KEGG) and Gene Ontology (GO) enrichment analyses showed that GSDMB and its interacting proteins might affect tumor growth through the serine metabolism pathway. Our current results demonstrate a promising therapeutic strategy targeting GSDMB and provide new insights into GSDMB as an immunological and prognostic biomarker for KIRC.

## Introduction

Cancer remains the leading cause of death worldwide and a challenging obstacle to improved life expectancy (1). Unfortunately, traditional chemotherapy options for cancers have significant side effects mitigated by immunotherapy-based approaches (2, 3). Over the past decade, immune checkpoint inhibitors (ICIs), especially the anti-PD-1/PD-L1 or anti-CTLA4 antibodies, have shown excellent prospects in many cancers. However, the tumor-infiltrating lymphocyte (TIL) level dramatically determines the efficacy of the immunotherapies overcoming tumor-driven immunosuppression (2). Interestingly, tumor-specific neoantigens and tumor mutagenicity are related to tumor immunogenicity, affecting the efficacy of the immune-checkpoint therapy (2, 3). In contrast, ICIs are ineffective in immune “cold” tumors with low lymphocyte infiltration.

Pyroptosis is lytic cell death and a critical effector pathway of innate immunity, which can promote “cold” tumor to “hot” tumor conversion (4). Historically, pyroptosis was initially defined as a caspase-1/4/5/11-mediated cell death with cytokine secretion (4). However, recently, the gasdermin (GSDM) family members were identified as the driving pore-forming effector proteins for membrane rupture and pyroptosis (5). The expression of GSDMs was closely related to many biological processes, including cancer-related pathways, drug resistance, immune subtype, and tumor microenvironment regulation (6). GSDMs comprise six human paralogous genes, GSDMA, GSDMB, GSDMC, GSDMD, GSDME (also known as DFNA5), and PJVK (also known as DFNB59), of which only GSDMB (or GSDML) is not found in rodents (5, 7). Gasdermins have an N-terminal pore-forming domain and a C-terminal inhibitory domain, which can be cleaved by caspase-1 or caspase-11/4/5 (8). Upon caspase cleavage, the N-terminal domain binds to membrane phospholipids and oligomerizes to form pores on the plasma membrane (9–16). In multiple cancer types, GSDM family genes tended to be upregulated compared with adjacent normal tissues, while there is no inherent uniform pattern in the expression of each GSDM gene (6). GSDME is cleaved and activated by caspase-3, similar to GSDMD by caspase-1/11 (5, 10, 16), and can switch caspase-3-driven apoptosis to pyroptosis, making cancer cells more sensitive to cytotoxic agents (17). However, all six GSDM members have marked intra- and inter-cancer heterogeneity concerning corresponding gene expression levels (6). Also, GSDMs are widely related to different cancers, making it impossible to determine whether a particular GSDMs gene is an oncogene or a tumor suppressor gene without selecting a cancer type (6).

The human GSDMB gene has six alternatively spliced transcripts and consists of 12 exons (18). GSDMB is involved in pyroptosis caused by granzyme A (GZMA) secreted by cytotoxic T lymphocytes (CTLs) and natural killer (NK) cells. GZMA predominantly cleaves at residue Lys244 within the interdomain linker to activate the GSDMB N-terminal leading to pore formation and release of pro-inflammatory cytokines (19, 20). Also, *Shigella flexneri* can secrete IpaH7.8 to ubiquitinate and target GSDMB for 26S proteasome degradation to evade natural killer cell responses (21). To further identify the potential functions of GSDMB, we analyzed the pan-cancer expression of GSDMB, including mRNA expression, clinical survival and prognosis, immune cell infiltration, and potential signal pathways from The Cancer Genome Atlas (TCGA) database to explore the potential mechanism of tumorigenesis and tumor inhibition among different cancer species (9, 22) (**Figure S1**).

Renal cell carcinoma (RCC) is the most common malignant kidney tumor, accounting for approximately 80%–90% of renal malignancies and 2%–3% of systemic malignant tumors (23). RCC consists of various pathological subtypes characterized by distinct genetic variants, histological changes, and various responses to therapies, including kidney renal clear cell carcinoma (KIRC) (~75%), kidney chromophobe (KICH, ~5%), and kidney renal papillary cell carcinoma (KIRP; 10%~16%) (24). However, approximately 15% of RCC patients, due to the lack of reliable and specific diagnostic biomarkers, progress to distant metastases at clinical diagnosis and have a poor prognosis (23, 25). Through continuous development over the past two decades, systemic therapy of RCC includes the VEGF-tyrosine kinase inhibitors or the anti-VEGF antibodies, mTOR pathway inhibition, and ICIs (26). Among them, the development of ICIs has transformed the management of advanced RCC. However, most RCC patients still do not experience durable clinical benefits due to susceptibility to drug resistance (27). Recently, GPX1 is reported as a biomarker for the diagnosis and prognosis of kidney cancer, while DDX1 may serve as a prognostic marker for renal cancer (23, 28). Nevertheless, due to the susceptibility to drug resistance of patients with advanced renal cancer, there is still an urgent need to discover specific diagnostic biomarkers and new therapeutic targets for RCC to improve the life quality of patients with advanced renal cancer (23).

## Results

### Clinical feature analysis and prognostic value of GSDMB in different cancers

**Figure 1:**
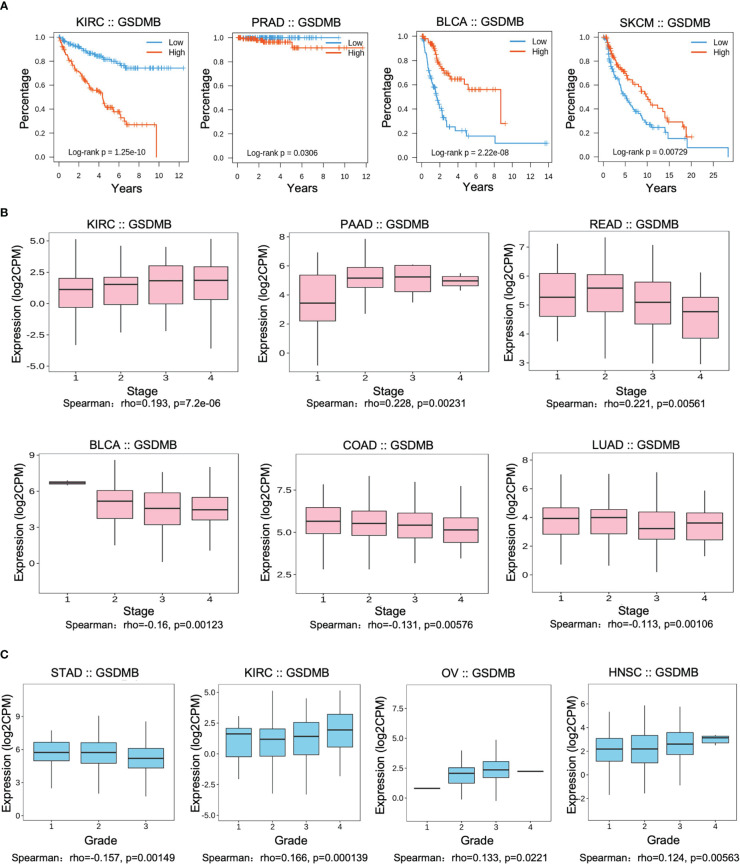
Associations between GSDMB and clinical features. **(A)** Associations between GSDMB expression and overall survival across human cancers. Kaplan–Meier survival curves for GSDMB gene expression were associated with overall survival. Patients were divided into high- and low-expression groups defined by the expression level of GSDMB gene (median was the cutoff). **(B)** Associations between GSDMB expression and stage across human cancers. Spearman’s correlation coefficient (rho) for GSDMB gene expression is positive in KIRC, PAAD, and rectal carcinoma (READ). Spearman’s correlation coefficient (rho) for GSDMB gene expression is negative in BLCA, COAD, and LUAD. **(C)** Associations between GSDMB expression and grade across human cancers. Spearman’s correlation coefficient (rho) for GSDMB gene expression is positive in KIRC, OV, and HNSC and negative in STAD. Spearman’s rank correlation rho represents the direction of association between X (independent variable) and Y (dependent variable). The magnitude of Spearman’s correlation increases as X and Y become closer to a perfect monotonic function of each other. When X and Y are entirely monotonically correlated, Spearman’s correlation coefficient becomes 1. GSDMB, gasdermin B; KIRC, kidney renal clear cell carcinoma; PAAD, pancreatic adenocarcinoma; READ, rectal carcinoma; BLCA, bladder urothelial carcinoma; COAD, colon adenocarcinoma; LUAD, lung adenocarcinoma; KIRC, kidney renal clear cell carcinoma; OV, ovarian serous cystadenocarcinoma; HNSC, head and neck squamous cell carcinoma; STAD, stomach adenocarcinoma.

To assess the relevance of GSDMB for clinical prognosis (**Figures 1**, **S2**), we analyzed the association between GSDMB expression and overall survival (OS), cancer stage, and tumor grade across different human cancers (**Figures 1A–C**, **S2**). Log-rank analysis showed that GSDMB gene expression in KIRC had the highest impact on OS (p < 0.0001) and, to a less extent, in prostate adenocarcinoma (PRAD) (p < 0.05). GSDMB gene is an unfavorable prognostic marker in KIRC and PRAD and a favorable prognostic marker in bladder urothelial carcinoma (BLCA) and skin cutaneous melanoma (SKCM) (**Figure 1A**). GSDMB has a positive protective effect on the prognosis of BLCA (p < 0.0001) and SKCM (p = 0.00729) tumor patients in BLCA tumors with a prognosis of approximately 6 years. Compared with patients with low expression of GSDMB, high expression of GSDMB can protect patient survival by more than 40%. However, in KIRC tumors, high expression of GSDMB has the opposite effect, reducing the 8-year survival rate of patients with a prognosis of 20% (**Figure 1A**). Additionally, the “Clinical” module of TISIDB was used to analyze the association between GSDMB and the clinical progression (stage) of different cancers (**Figures 1B, C**). Results showed that the expression of GSDMB has a significant positive relationship with the disease stage of KIRC tumor patients (p < 0.0001). Also, there is a high expression of GSDMB in advanced KIRC tumors and high-grade KIRC tumors (p = 0.000139) (**Figures 1B, C**). Contrastingly, low expression of the GSDMB was associated with poor OS and prognosis for BLCA (p < 0.0001) and SKCM (p = 0.00729) (**Figures 1A**, **S3**). Further analysis of the relationship between the BLCA tumor stage and GSDMB expression showed that the expression of GSDMB was inhibited in advanced BLCA tumors (p = 0.00123), which is consistent with the clinical prognostic data.

### Promoter methylation analysis of GSDMB

**Figure 2:**
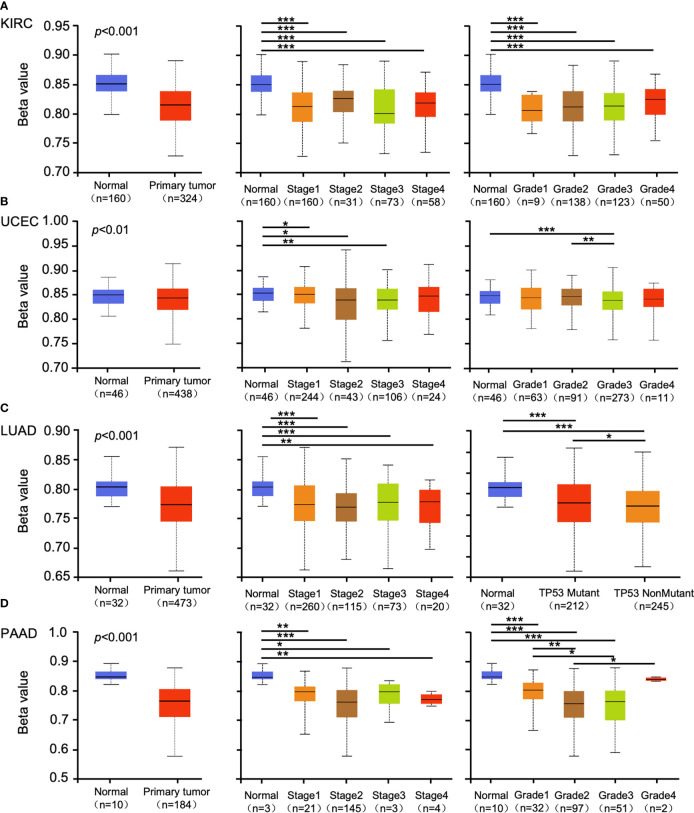
The promoter methylation level of GSDMB in different cancers. The promoter methylation of the GSDMB in KIRC **(A)**, UCEC **(B)**, LUAD **(C)**, and PAAD **(D)** with different stages, grades, and TP53 mutant pathways were analyzed through the UALCAN from the CPTAC dataset. Based on GSDMB promoter methylation profiles by sample types, promoter methylation levels of GSDMB were significantly reduced in KIRC, UCEC, LUAD, and PAAD primary tumors compared with normal. Based on GSDMB promoter methylation profiles by individual cancer stages/tumor grade, promoter methylation levels of GSDMB were significantly reduced in KIRC, UCEC, LUAD, and PAAD. The beta value indicates DNA methylation level ranging from 0 (unmethylated) to 1 (fully methylated). Different beta value cutoff has been considered to indicate hyper-methylation [beta value: 0.7–0.5] or hypo-methylation [beta value: 0.3–0.25]. *p < 0.05; **p < 0.01; ***p < 0.001. GSDMB, gasdermin B; KIRC, kidney renal clear cell carcinoma; UCEC, uterine corpus endometrial carcinoma; LUAD, lung adenocarcinoma; PAAD, pancreatic adenocarcinoma; CPTAC, Clinical Proteomic Tumor Analysis Consortium.

**Figure 3:**
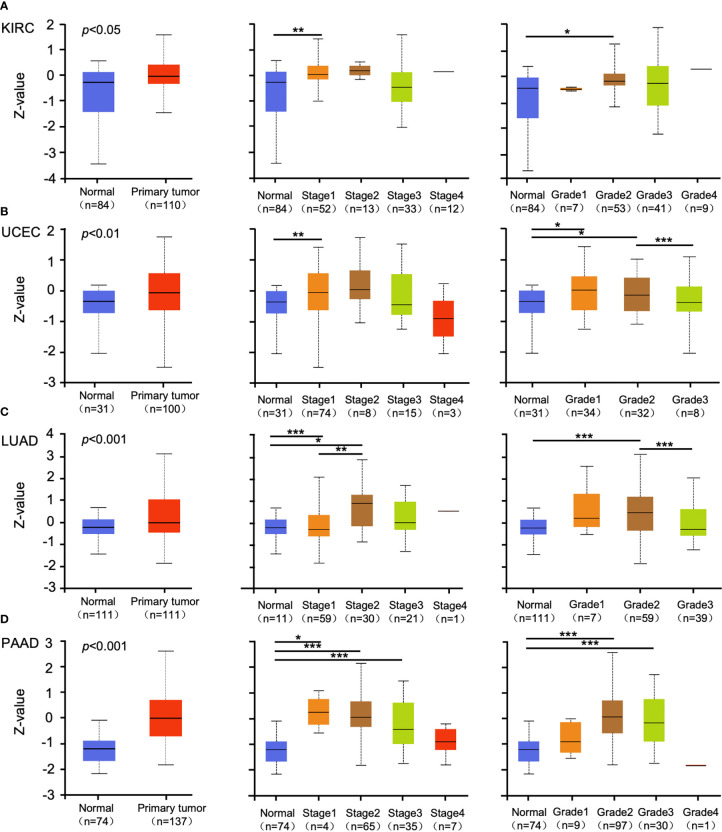
The protein expression levels of GSDMB in different cancers. **(A–D)** The expression levels of the GSDMB protein between normal tissue and primary tissue of KIRC **(A)**, UCEC **(B)**, LUAD **(C)**, and PAAD **(D)** with different stages and grades were analyzed through the UALCAN from the CPTAC dataset. Based on GSDMB proteomic expression profiles of sample types, GSDMB protein expression was significantly increased in KIRC, UCEC, LUAD, and PAAD primary tumors compared with normal tissue. Based on GSDMB proteomic expression profiles of individual cancer stages, GSDMB protein expression was significantly increased in the KIRC cancer stage 1, UCEC cancer stage 1, LUAD cancer stage 1, LUAD cancer stage 2, PAAD cancer stage 1, PAAD cancer stage 2, and PAAD cancer stage 3 compared with normal. Based on GSDMB proteomic expression profiles of tumor grade, GSDMB protein expression was significantly increased in KIRC tumor grade 2, UCEC tumor grade 1, UCEC tumor grade 2, LUAD tumor grade 2, PAAD tumor grade 2, and PAAD tumor grade 3. Z-values represent standard deviations from the median across samples for the given cancer types. *p < 0.05; **p < 0.01; ***p < 0.001. GSDMB, gasdermin B; KIRC, kidney renal clear cell carcinoma; UCEC, uterine corpus endometrial carcinoma; LUAD, lung adenocarcinoma; PAAD, pancreatic adenocarcinoma; CPTAC, Clinical Proteomic Tumor Analysis Consortium.

Promoter methylation regulates gene expression in different cancers (29, 30). Methylation analysis showed that promoter methylation negatively correlated with GSDMB expression level in KIRC (p < 0.001), uterine corpus endometrial carcinoma (UCEC) (p < 0.01), lung adenocarcinoma (LUAD) (p < 0.001), and pancreatic adenocarcinoma (PAAD) (p < 0.001) (**Figures 2A–D**). The promoter methylation level of GSDMB negatively regulated the degree of tumor development and tumor differentiation of KIRC (**Figure 2A**). Also, it negatively regulated UCEC tumor development stage 1, stage 2 and stage 3, and differentiation grade 3 (**Figure 2B**) and significantly negatively regulated the progression of LUAD tumors, in which tumor onset was associated with TP53 mutation (p < 0.05) (**Figure 2C**). Moreover, GSDMB was negatively regulated in the progression and differentiation of PAAD tumors, primarily stage 2 (p < 0.001) and grades 1 to 3 (**Figure 2D**). The promoter methylation levels of GSDMB have distinct effects on protein expression in different cancers. Surprisingly, high promoter methylation levels of GSDMB are related to poor prognosis of KIRC patients, similar to UCEC, LUAD, and PAAD, which showed contrasting clinical outcomes based on GSDMB expression. Meanwhile, at the protein level and compared with normal tissues, GSDMB protein expression was higher in KIRC, UCEC, LUAD, and PAAD (**Figures 3A–D**, **S4B**). The methylation level of the promoter regulates the protein level of GSDMB, and the analysis results also support this conclusion.

### Tumor–immune system interactions analysis

**Figure 4:**
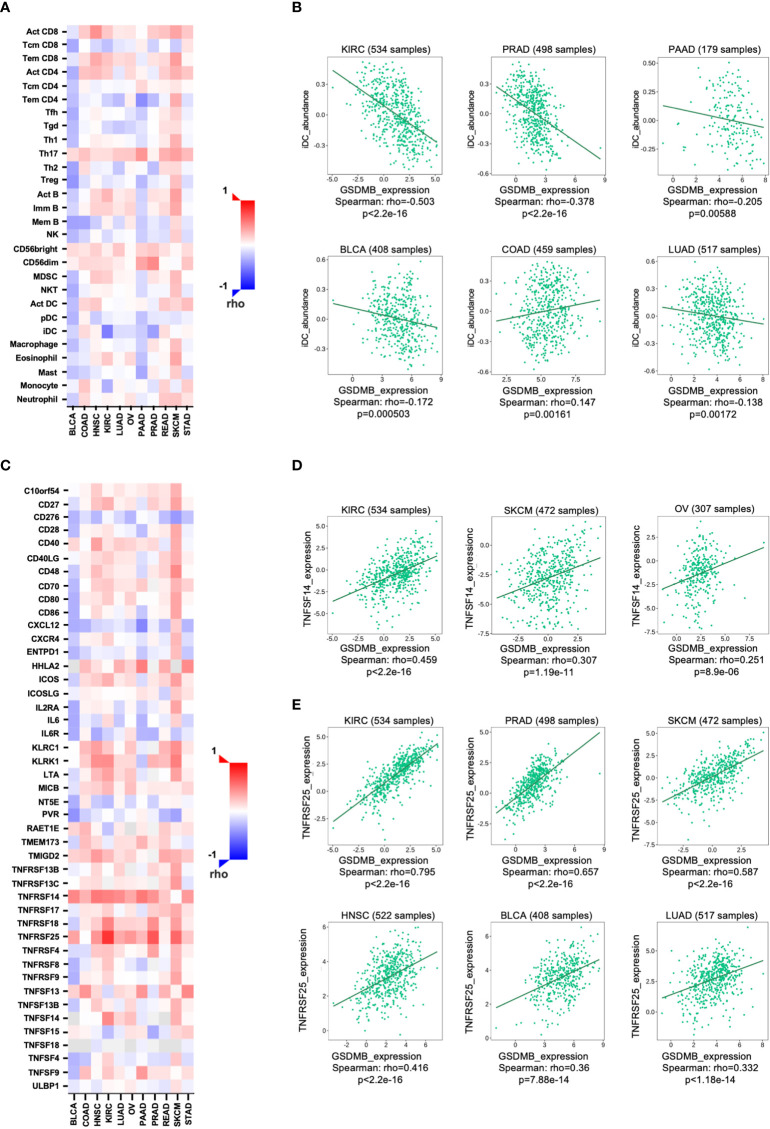
The relationship between GSDMB expression and two kinds of immunomodulators. **(A)** Spearman’s correlations between GSDMB and TILs across human cancers. **(B)** Representative correlation dot plots for iDCs. The expression of GSDMB gene in KIRC has the most significant negative correlation with iDCs. **(C)** Spearman’s correlations between GSDMB and immunostimulators across human cancers. Representative correlation dot plots for TNFSF14 **(D)** and TNFRSF25 **(E)**. The expression of GSDMB gene in KIRC has the most significant positive correlation with TNFSF14/TNFRSF25. Spearman’s correlations: the value in the following heatmap represents the rho value. GSDMB, gasdermin B; TILs, tumor-infiltrating lymphocytes; iDCs, immature dendritic cells; KIRC, kidney renal clear cell carcinoma.

We next investigated the relationship between the abundance of TILs and the expression, copy number, methylation, and mutation of GSDMB by using the database containing 28 TIL types of immune-related characteristics from Charoentong’s study (31, 32). For each cancer type, the relative abundance of TILs was inferred by the gene set variation analysis (GSVA) based on the gene expression profile. The analysis results show that in KIRC (rho = −0.503) and PRAD (rho = −0.378), but not in other cancers, immature dendritic cells (iDCs) may be regulated by GSDMB gene (**Figures 4A, B**; **S5**). iDCs are generally associated with scouting functions through endocytosis but can differentiate into mature DCs through pattern recognition receptor (PRR) simulation and contribute to antigen presentation functions (33). Therefore, DCs are dominant partners of T cells, indispensable for initiating adaptive immune responses (34). The high expression of GSDMB in KIRC is related to the low expression levels of iDCs in TILs, which may be one of the fundamental reasons for the poor clinical prognosis of KIRC patients with high GSDMB expression (**Figure 1A**). However, the level of TILs in BLCA patients with high GSDMB expression is generally low, which may reveal why the clinical prognosis of this type of BLCA patient has been abysmal for more than 8 years (**Figure 1A**). This is a long-term slow effect on the combat effectiveness of the systemic immune system.

### GSDMB regulates immunomodulators in KIRC

To unveil the relationship between different immunomodulators and GSDMB expression, copy number, methylation, or mutation, 534 samples in the TISIDB database were analyzed using Spearman’s correlation test. Two immunomodulators, TNFSF14 and TNFRSF25, were found to regulate GSDMB gene expression in cancers (**Figures 4C, D**). TNFSF14 is positively correlated with GSDMB in KIRC (rho = 0.459, p < 0.0001), SKCM (rho = 0.307, p < 0.0001), and ovarian serous cystadenocarcinoma (OV) (rho = 0.251, p < 0.0001) patients (**Figures 4C, D**). Meanwhile, TNFRSF25 is positively correlated with GSDMB expression in KIRC (rho = 0.795, p < 0.0001), PRAD (rho = 0.657, p < 0.0001), SKCM (rho = 0.587, p < 0.0001), head and neck squamous cell carcinoma (HNSC) (rho = 0.416, p < 0.0001), BLCA (rho = 0.36, p < 0.0001), and LUAD (rho = 0.332, p < 0.0001) as well (**Figures 4C, E**). The two immunomodulators (TNFSF14 and TNFRSF25) were consistently highly expressed in KIRC (collected from Charoentong’s study) and were therefore selected for further analysis.

TNFSF14 can activate TNFRSF3/LTBR as a costimulatory signal for T-cell proliferation and interferon-γ (IFN-γ) production; however, this pathway is regulated by the decoy receptor TNFRSF6B and the interaction with TNFRSF14/HVEM (35). Previous reports have demonstrated that IFN-γ can induce the protein expression of GSDMB in cells (19). Combined with our analysis results, IFN-γ and GSDMB are likely to form a positive closed loop through TNFSF14. TNFRSF25 is the receptor of TNFSF12/APO3L/TWEAK, which regulates lymphocyte homeostasis and mediates direct interaction with the adapter protein TRADD to initiate NF-κB activation and induce apoptosis (36–38). Notably, TNFRSF25 was prevalently and significantly positively correlated with high GSDMB expression in different tumors (**Figures 4C, E**). The results suggest that the role of GSDMB gene may be involved in the pathway of NF-κB activation and induction of apoptosis related to TNFRSF25 and TRADD.

### GSDMB-related immune infiltration analysis

**Figure 5:**
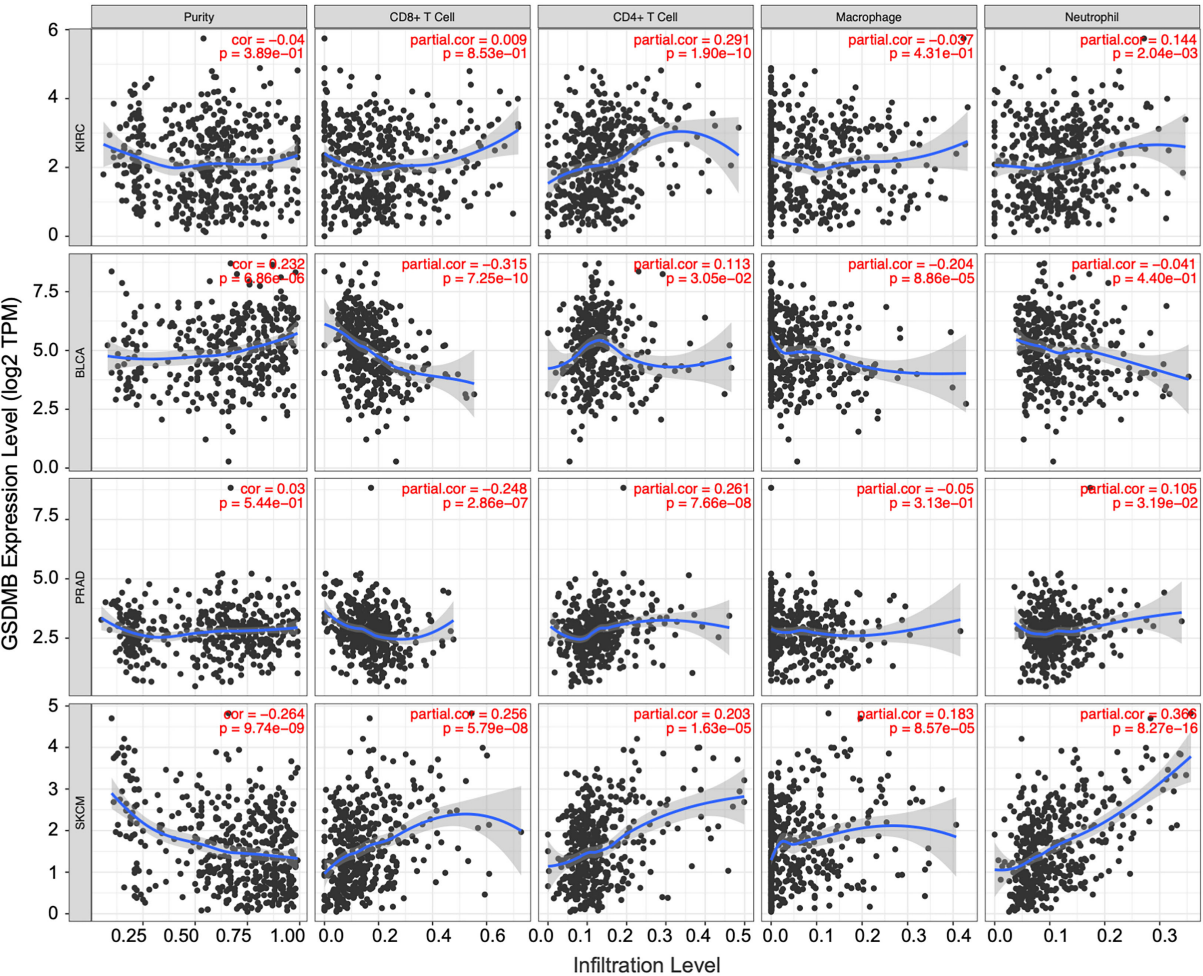
The correlation between GSDMB expression and immune infiltration levels in diverse cancer types. Generated and displayed scatterplots showing the purity-corrected partial Spearman’s rho value and statistical significance. Gene expression levels for tumor purity are consistently shown on the leftmost panel. The scatter plots of related cancers generated by Spearman’s algorithm, including KIRC, BLCA, PRAD, and SKCM. In KIRC, the expression level of GSDMB gene was significantly and positively correlated with CD4+ T cells. GSDMB, gasdermin B; KIRC, kidney renal clear cell carcinoma; BLCA, bladder urothelial carcinoma; PRAD, prostate adenocarcinoma; SKCM, skin cutaneous melanoma.

The tumor microenvironment (TME) is complex and dynamic, with various types of cells, including endothelial cells, fibroblasts, immune cells, and stromal cells (39). Mechanistically, cytokines in TME modulate immune function and can lead to weak immune response and tumor progression (39). In addition, immunosuppressive TME is associated with tumor occurrence, growth, and metastasis (40). TILs and TME are vital factors for tumor growth and disease progression. Therefore, we assessed the potential correlation between GSDMB expression and immune cell infiltration in different cancers (**Figures 5**, **S6**). CD4+ T lymphocytes, responsible for eliminating and controlling various infections, in KIRC patients (partial.cor = 0.291, p < 0.0001) were positively correlated with the expression of GSDMB. When a naive CD4+ T cell is activated by antigen-presenting cells, it can develop into several different functional T helper (T_H_) cells. The immune response activity of CD4+ TH1 cells is mainly mediated by the production of its hallmark cytokine IFN-γ (41). Therefore, GSDMB can be induced by IFN-γ.

Similarly, other TILs can be used as auxiliary judgment indicators for clinical disease diagnosis and prognosis. For example, in the BLCA tumor microenvironment with high GSDMB expression, the infiltration levels of CD8+ T cells (partial.cor = −0.315, p < 0.0001), macrophages (partial.cor = −0.204, p < 0.0001), and dendritic cells (partial.cor = −0.215, p < 0.0001) were low; in the same PRAD tumor microenvironment with high GSDMB expression, the infiltration level of CD8+ T cells (partial.cor = −0.248, p < 0.0001) was low, and the infiltration level of CD4+ T cells (partial.cor = 0.261, p < 0.0001) was high; common immune-infiltrating lymphocytes generally existed at high levels in the SKCM tumor microenvironment with high GSDMB expression (**Figure 5**).

### Enrichment analysis for GSDMB-related genes

**Figure 6:**
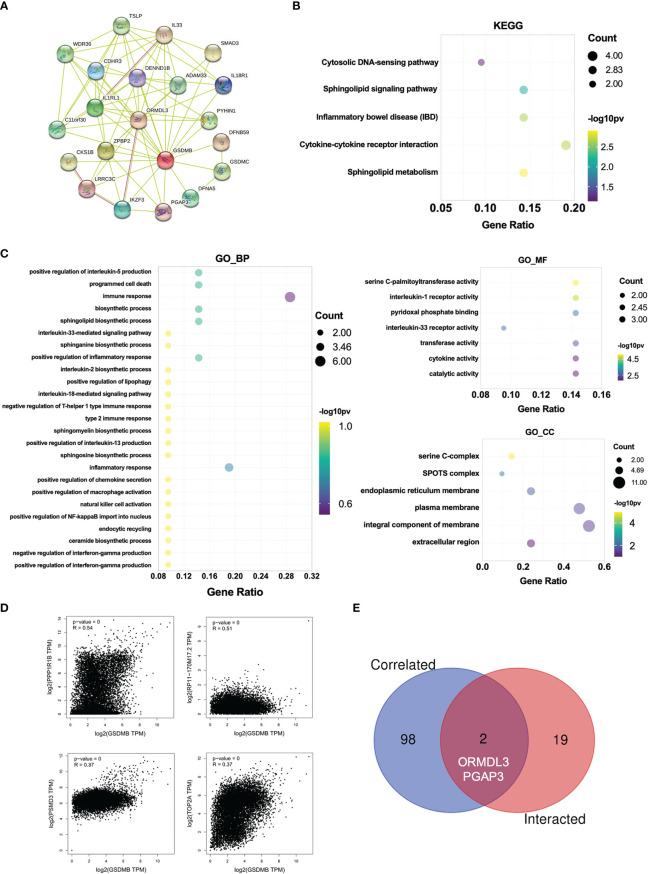
Enrichment analysis for GSDMB-related genes. **(A)** Protein–protein interaction network between all available experimentally determined GSDMB-binding proteins using the STRING tool. **(B)** The KEGG pathway and GO enrichment analyses **(C)** were performed on GSDMB-binding genes. **(D)** The correlation between GSDMB and selected binding genes, such as PPP1R1B, RP11-170M17.2, PSMD3, and TOP2A. **(E)** The Venn diagram shows two types of crossover genes, including ORMDL3 and PGAP3. GSDMB, gasdermin B; KEGG, Kyoto Encyclopedia of Genes and Genomes; GO, Gene Ontology.

Next, to investigate the potential mechanisms that GSDMB participated in the progression of cancers, we used the online tool STRING to construct a protein–protein interaction (PPI) network for GSDMB (**Figure 6A**). The DAVID tool was used to perform Kyoto Encyclopedia of Genes and Genomes (KEGG) and Gene Ontology (GO) enrichment analyses across many cancer types. The results demonstrate that GSDMB interacted protein was significantly involved in cytokine–cytokine receptor interaction, sphingolipid metabolism, inflammatory bowel disease (IBD), and several immune pathways such as IL18/33 production, IFN-γ production, and inflammatory response (**Figures 6B, C**). Based on biological process (BP) analysis, GSDMB may participate in immune response, programmed cell death, biosynthetic process, and other pathways to regulate tumor progression. The cellular component (CC) analysis shows these genes mainly exist in the serine C-complex, plasma membrane, and integral component of the membrane. Based on molecular function (MF) analysis, these genes have serine C-palmitoyltransferase activity, cytokine activity, transferase activity, and catalytic activity (**Figure 6C**). It is worth mentioning that abnormal sphingolipid metabolism is often associated with cancer and neurodegenerative diseases. In addition, serine palmitoyltransferase affects tumor growth by regulating phospholipid metabolism (42). Moreover, we evaluated the correlation between GSDMB and selected expression-related genes, such as PPP1R1B, RP11-170M17.2, PSMD3, and TOP2A (**Figure 6D**). Two types of crossover genes (ORMDL3 and PGAP3) were predicted to have a significant association with GSDMB (**Figure 6E**). These results indicate that GSDMB plays an essential role in cancer metabolism and immunity.

### Genetic alteration analysis

**Figure 7:**
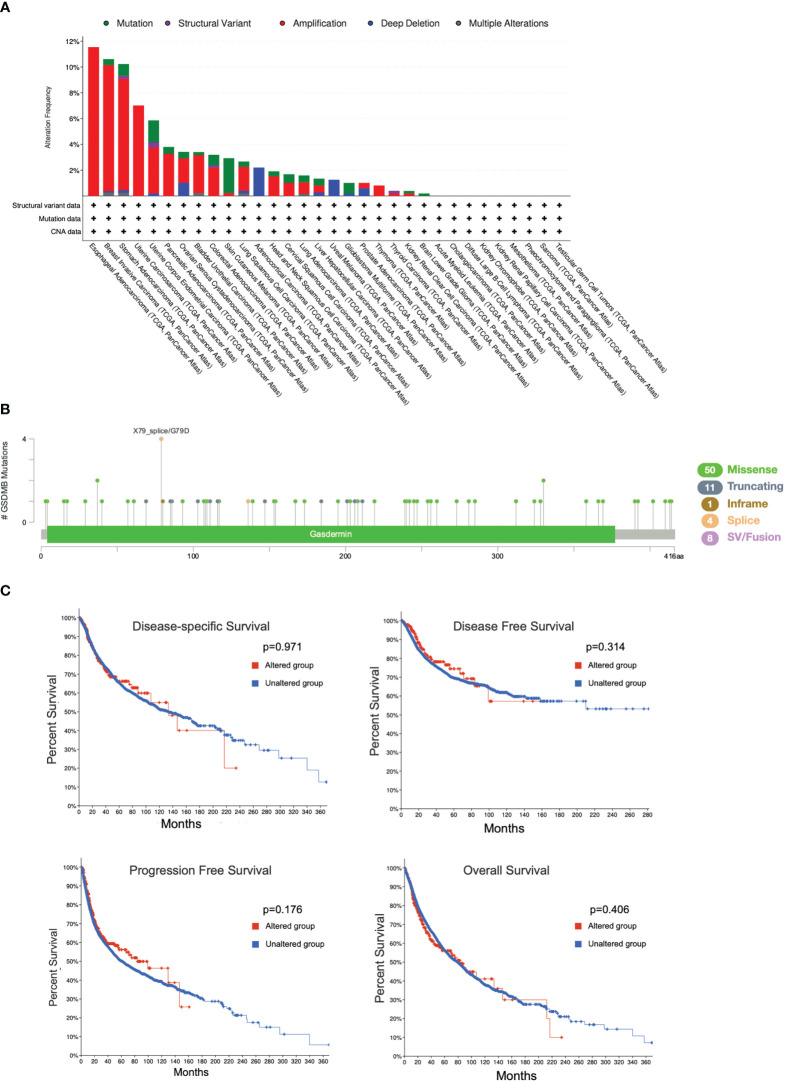
Mutation features of GSDMB in different cancers in TCGA. The alteration frequencies with mutation type **(A)** and mutation site **(B)** are shown. Each color denotes a mutation type. Mutation (green): point mutation. **(C)** The potential correlation between the mutation status of GSDMB and overall survival, disease-free survival, progression-free survival, and disease-specific survival of different tumors using the cBioPortal tool. GSDMB, gasdermin B; TCGA, The Cancer Genome Atlas.

To reveal whether the genetic alterations of GSDMB affect tumor occurrence and the prognosis of patients, the cBioPortal web (https://www.cbioportal.org/) was used to analyze the data from the TCGA. Among the different types of genetic alterations, amplification was the most dominant type, especially in the esophageal adenocarcinoma (EAC) (11.54% of whole cases) and breast invasive carcinoma (BRCA) types (9.78% of whole cases) (**Figure 7A**). In addition, GSDMB mutation frequencies are the highest in SKCM and UCEC, whereas the missense mutation is the most common type of GSDMB mutation, with a total of 50 cases of missense type, accounting for 67.5% (50/74) (**Figures 7A, B**). We also investigated the potential relationship between GSDMB genetic alterations and prognosis in patients with multiple cancer types. However, as shown in **Figure 7C**, mutated GSDMB revealed no association with disease-free survival (p = 0.314), disease-specific survival (p = 0.971), overall survival (p = 0.406), and progression-free survival (p = 0.176), as compared with species without GSDMB alternation. Taken together, no potential association was found between the genetic alteration of GSDMB and the clinical survival prognosis of different cancers.

## Discussion

Increasing evidence shows that GSDMB is highly expressed in some tissues and can be upregulated by IFN-γ, involved in the development and prognosis of multiple cancers (5, 6). In addition, GZMA from cytotoxic lymphocytes can cleave and activate GSDMB to induce pyroptosis in tumor cells as well as protect host cells during viral infection and prevent evasion from natural killer cell immunity (5). Therefore, GSDMB may function in tumor suppression. T-cell exhaustion, depletion of CD8+ cells in a large proportion, and abnormal metabolic patterns coinciding in cancer cells and tumor-infiltrating stromal cells are key factors leading to the immunosuppressive property of clear cell renal cell carcinoma (ccRCC) tissue, which is closely related to poor prognosis (43). Previous studies reported that MMP25-AS1/hsa-miR-10a-5p/SERPINE1 axis is a new prognostic biomarker related to the infiltration of immune cells in KIRC (44), tumor antigen, and immune subtype, guiding the development of mRNA vaccine for clearing renal cell carcinoma (45). In addition, iron-related gene CHAC1 effectively indicates a poor prognosis of renal clear cell carcinoma (46). However, few genes related to the death pathway of pyroptosis have been reported. Here, we conducted a comprehensive screening of GSDMB expression, genetic alteration, immune infiltration, and possible related pathways in previously published pan-cancer databases. The potential oncogenic roles of GSDMB across 33 tumors based on the TCGA and the Genotype-Tissue Expression (GTEx) databases were explored, and the results revealed that GSDMB is highly expressed in many tumors, including KIRC, UCEC, LUAD, and PAAD (**Figure 2**). Based on the Gene Expression Omnibus (GEO) database, the RNA expression level of GSDMB in ccRCC and its paracancerous samples were compared (GSE40435, N = 101). The results showed that the RNA level of GSDMB in ccRCC was significantly higher than that in the paracancerous samples (**Figure S4A**). In particular, high GSDMB expression was significantly related to the poor prognosis of KIRC patients, whereas low GSDMB expression was associated with poor prognosis of BLCA and SKCM patients (**Figure 2A**).

Although the prognostic relevance of GSDMB in ccRCC has been mentioned (46), our further immune infiltration analysis revealed that the level of CD4+ T-cell infiltration in tumors was influenced by GSDMB expression and significantly correlated with iDCs in KIRC (**Figures 4A, B**, **5**). The abundance of iDCs in TILs was negatively correlated with GSDMB expression, and the number of infiltrating DC in most solid tumors was positively correlated with the patient’s prognosis. Therefore, the negative correlation between GSDMB expression and iDC abundance may be one of the essential factors for the poor prognosis of KIRC patients. These results suggested that GSDMB may be a prognostic marker of RCC and a clinical diagnostic marker of KIRC. As GSDMB has become a new therapeutic target for cancer in combination with different immunotherapies, this study suggests that it might be especially efficacious in KIRC patients.

Moreover, the immunomodulators TNFRSF25 and TNFSF14 were significantly positively correlated with GSDMB expression in many tumors (**Figures 4C–E** and **S5**). TNFRSF25 directly interacts with the adaptor TRADD to mediate NF-κB activation and induce apoptosis (36–38). TNFSF14 acts as a TNFRSF14/HVEM receptor and transmits costimulatory signals to T cells, leading to T-cell proliferation and IFN-γ production, which enriches the previous report that IFN-γ induces GSDMB protein production in cells (35). Lastly, the KEGG and GO enrichment analyses on multiple cancer types showed that GSDMB could bind to membrane lipids such as phosphatidylinositol(4,5)bisphosphate, phosphatidylinositol 5-phosphate, and bisphosphorylated phosphatidylinositols and weakly bind phosphatidic acid (20) (**Figure 6**). Also, the GSDMB interacted proteins were mainly present in the serine C-complex, plasma membrane, and integral component of the membrane (**Figure 6C**). GSDMB is significantly involved in many signal pathways, including cytokine–cytokine receptor interaction, sphingolipid metabolism, IBD, and other immune pathways (**Figure 6B**), which are implicated in cancer and neurodegenerative diseases (47). Moreover, asthma susceptibility gene ORMDL3 promotes autophagy of human bronchial epithelium (48). Therefore, GSDMB, PGAP3, and ORMDL3 are the leading candidate asthma genes (49). Given the importance of epithelial integrity in asthma, we assume that ORMDL3 and PGAP3 directly affect the function of renal epithelial cells.

Pan-cancer analysis has been successfully used to identify different diagnostic and prognostic markers in many cancers (48–52). However, despite extensive efforts, there are still some limitations due to the lack of relevant data from cell and animal experiments. Our results indicated that GSDMB expression is associated with tumor immunity and clinical survival prognosis. Unfortunately, we cannot assert if GSDMB could affect clinical survival *via* a defined signaling pathway. However, we demonstrate a promising therapeutic strategy targeting GSDMB, which may improve KIRC patient prognosis, provide an understanding of the possible underlying mechanisms of action, and provide new insights into GSDMB as a prognostic marker and potential therapeutic target for cancers.

## Materials and methods

### Raw data acquisition and processing

We input GSDMB into the “Diff Exp” module of the TIMER website (https://cistrome.shinyapps.io/timer/) (53, 54). Next, we studied the differential expression of GSDMB between the tumor and adjacent normal tissues in all TCGA tumors. The distribution of gene expression levels is shown in box plots, and the Wilcoxon test evaluates the statistical significance of differential expression. For each cancer type, we identify GSDMB upregulated or downregulated in the tumors compared with normal tissues for each cancer type, which are displayed in the gray column when standard data are available. Next, we entered GSDMB into the “Gene_DE” module of the TIMER2 website (http://timer.cistrome.org/). Then, we scanned the GSDMB expression difference between malignant cancers and corresponding normal tissues for diverse cancers and specific subtypes. To solve the imbalance between the tumor and standard data, which can cause inefficiency in various differential analyses, TCGA and GTEx gene expression data were available from the Gene Expression Profiling Interactive Analysis (GEPIA) web server (http://gepia.cancer-pku.cn/#analysis), which are re-computed from raw RNA-Seq data by the UCSC Xena project based on a uniform pipeline, thus allowing for the formation of the most comprehensive expression information. Herein, for specific cancers with limited normal and without normal tissues, we applied the “Expression analysis-BoxPlot” module and clicked the “Match TCGA normal and GTEx data” module of GEPIA to analyze the GSDMB level between the malignant cancers and adjacent normal tissues.

### Survival analysis

OS and disease-free survival (DFS) information of GSDMB within all TCGA cancers were obtained from the “Survival Map” module of GEPIA2. Cutoff-high (50%) and cutoff-low (50%) values were utilized as thresholds to stratify high- and low-expression cases. In addition, a log-rank test was used for the hypothesis test, and survival curves were graphed with the “Survival Analysis” module.

### GSDMB-related tumor–immune system interaction analysis

The tumor and immune system data of GSDMB were queried from TISIDB algorithms (http://cis.hku.hk/TISIDB/index.php). The information on GSDMB expression, copy number alteration (CNA), methylation (met), and mutation (mut) was visualized with the “Lymphocyte, Immunomodulator, Chemokine” module. In addition, the associations between GSDMB expression and overall survival, stage, and grade across human cancers were visualized with the “Clinical” module.

### The promoter methylation level of GSDMB analysis

The promoter methylation level of GSDMB was analyzed with the University of Alabama at Birmingham cancer data analysis portal (UALCAN) website (http://ualcan.path.uab.edu/index.html) (53, 54). UALCAN is a comprehensive, user-friendly, and interactive web resource for analyzing cancer omics data (TCGA, MET500, CPTAC, and CBTTC) (55). We inputted “GSDMB” in the “TCGA” module of UALCAN web; it provides “expression”, “survival”, “methylation”, “correlation”, and “pan-cancer view” analysis information with different cancers. GSDMB promoter methylation profiles were analyzed based on “sample types”, “individual cancer stages”, “tumor grade”, and “TP53 mutation status”, on the selected cancers KIRC, UCEC, LUAD, and PAAD. The different cutoff value for beta value has been considered to indicate hyper-methylation [beta value: 0.7–0.5] or hypo-methylation [beta value: 0.3–0.25] (56).

### Genetic alteration analysis of GSDMB

The genetic alteration features of GSDMB were queried from the “TCGA Pan Cancer Atlas Studies” module of the cBioPortal web (https://www.cbioportal.org/) (54). The alteration frequency, mutation type, and CNA information were visualized with the “Cancer Types Summary” module. “Mutations” modules obtained mutation types and frequencies of GSDMB at different positions. The potential correlation between the mutation alteration of GSDMB and disease-free survival, overall survival, progression-free survival, and disease-specific survival of different tumors were analyzed using the “Comparison/Survival” modules.

### GSDMB-related comprehensive analysis of tumor-infiltrating immune cells

The GSDMB-related immune infiltration-relevant results were analyzed with the TIMER pipeline (https://cistrome.shinyapps.io/timer/), a comprehensive resource for the systematic analysis of immune infiltrates across diverse cancer types. The abundances of six immune infiltrates (B cells, CD4+ T cells, CD8+ T cells, neutrophils, macrophages, and dendritic cells) were estimated by TIMER algorithm, which allows for user-generated function-specific parameters and dynamically displays the result map to easily access the tumor immunological, clinical, and genomic features. In addition, GSDMB expression data from different cancer types and immune infiltrates were uploaded into the “Gene” module of the TIMER website to generate scatterplots showing the purity-corrected partial Spearman’s rho value and statistical significance.

### GSDMB-related gene enrichment analysis

GSDMB-binding proteins were downloaded from STRING (https://string-db.org/). The top 100 GSDMB-binding genes were selected from analysis in TCGA of all cancers and adjacent normal tissues *via* the “Similar Gene Detection” module. Then, the “correlation analysis” module was used for pairwise gene Pearson’s correlation analysis with GSDMB. The “Gene_Corr” module was used to visualize the heatmap information of these selected genes. Finally, this gene list was uploaded to the Database for Annotation, Visualization and Integrated Discovery (DAVID) website to perform the Gene Ontology enrichment and KEGG pathway analyses. The results with p < 0.05 were considered statistically significant.

### Statistical data

All the data of gene expression were normalized by log2 transformation. Differences between normal and cancer tissues were assessed by the Wilcoxon rank-sum test. Correlation analysis between two variables was determined with Spearman’s or Pearson’s test. Survival analysis, hazard ratios (HRs), and p-values were calculated by the univariate Cox regression analysis or log-rank test. The Kaplan–Meier curves were used to compare the survival of patients stratified according to different levels of each chemokine expression. p < 0.05 was set as the significance threshold for all statistical analyses.

## Data availability statement

The datasets presented in this study can be found in online repositories. The names of the repository/repositories and accession number(s) can be found in the article/**Supplementary Material**.

## Author contributions

JL and SL conceived and designed the study. XL, FX and JD performed the experiments and analyzed the data. JL analyzed the data and wrote the manuscript. All authors contributed to the article and approved the submitted version.
