# Supplementary material for: Systematic pan-cancer analysis identifies gasdermin B as an immunological and prognostic biomarker for kidney renal clear cell carcinoma
Source: Front Oncol. 2023 Mar 30;13:1164214. doi: 10.3389/fonc.2023.1164214 (PMC10101337; doi:10.3389/fonc.2023.1164214)
Supplement: Supplementary file 1 [file DataSheet_1.pdf]

Systematic Pan-Cancer Analysis Identifies Gasdermin B as an Immunological and Prognostic Biomarker for Kidney Renal Clear Cell Carcinoma

Xuehe Liu[1,#], Feiyan Xie[1,#], Jin Ding[2], Suhua Li[3,*], Jixi Li[1,2,*]

[1]State Key Laboratory of Genetic Engineering, School of Life Sciences and Huashan Hospital, Shanghai Engineering Research Center of Industrial Microorganisms, MOE Engineering Research Center of Gene Technology, Fudan University, Shanghai, 200438, China.

[2]Clinical Cancer Institute, Center for Translational Medicine, Naval Medical University, Shanghai 200433, China.

[3]Division of Natural Science, Duke Kunshan University, Jiangsu, 215316, China.

[#] X. L. and F. X. contributed equally to this work.

*To whom correspondence should be addressed. Email: lijixi@fudan.edu.cn or suhua.li@dukekunshan.edu.cn

Supplementary materials

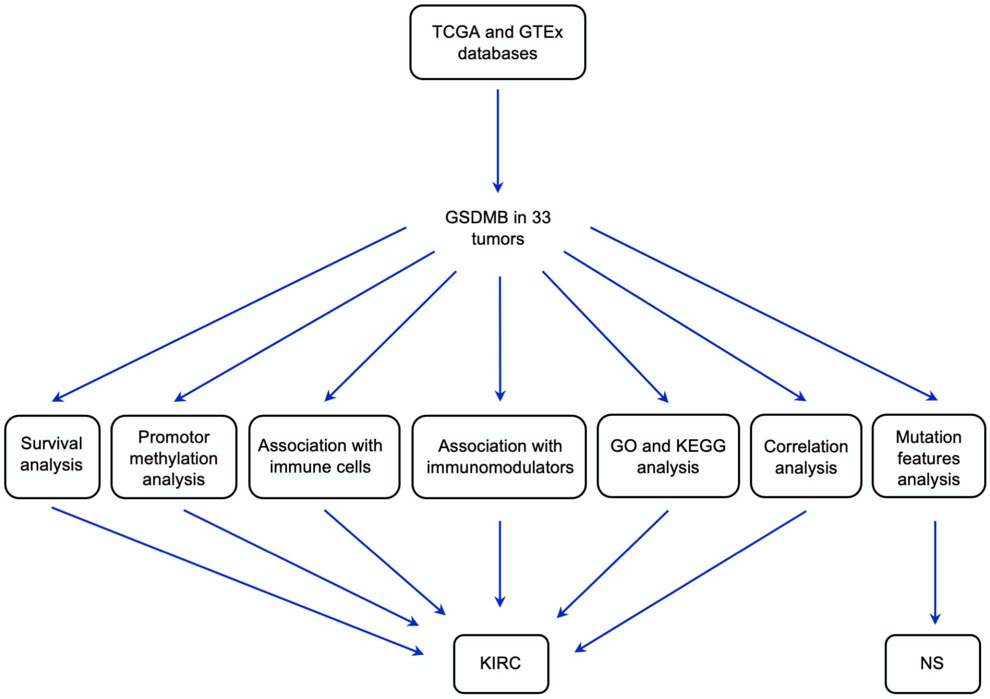

Figure S1. Bioinformatics flowchart of methodical analysis.

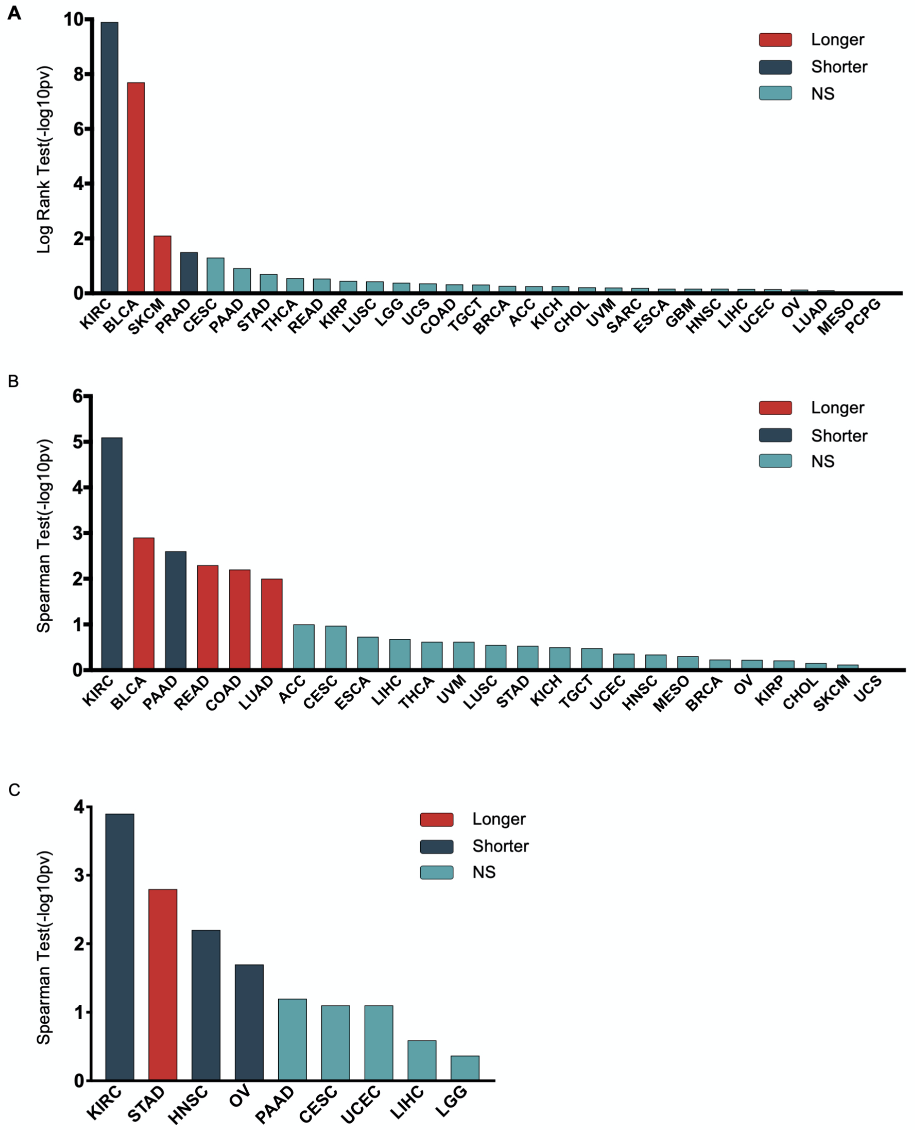

Figure S2. Associations between GSDMB and clinical features. Associations between GSDMB expression and clinical features in overall survival analysis (A), cancer stage (B), and tumor grade (C) across human cancers by column graphs. The GSDMB gene was most significantly associated with KIRC regarding overall survival analysis, cancer stage, and tumor grade. (A) The X axis longer (or shorter) showed that the GSDMB gene is associated with longer (or shorter) survival (Log-rank test: $p < 0.05$). (B) The X-axis Lower (or Higher)

showed that the GSDMB gene is associated with Lower (or Higher) stage/grade (C) Spearman correlation test: $p < 0.05$. NS: No significance.

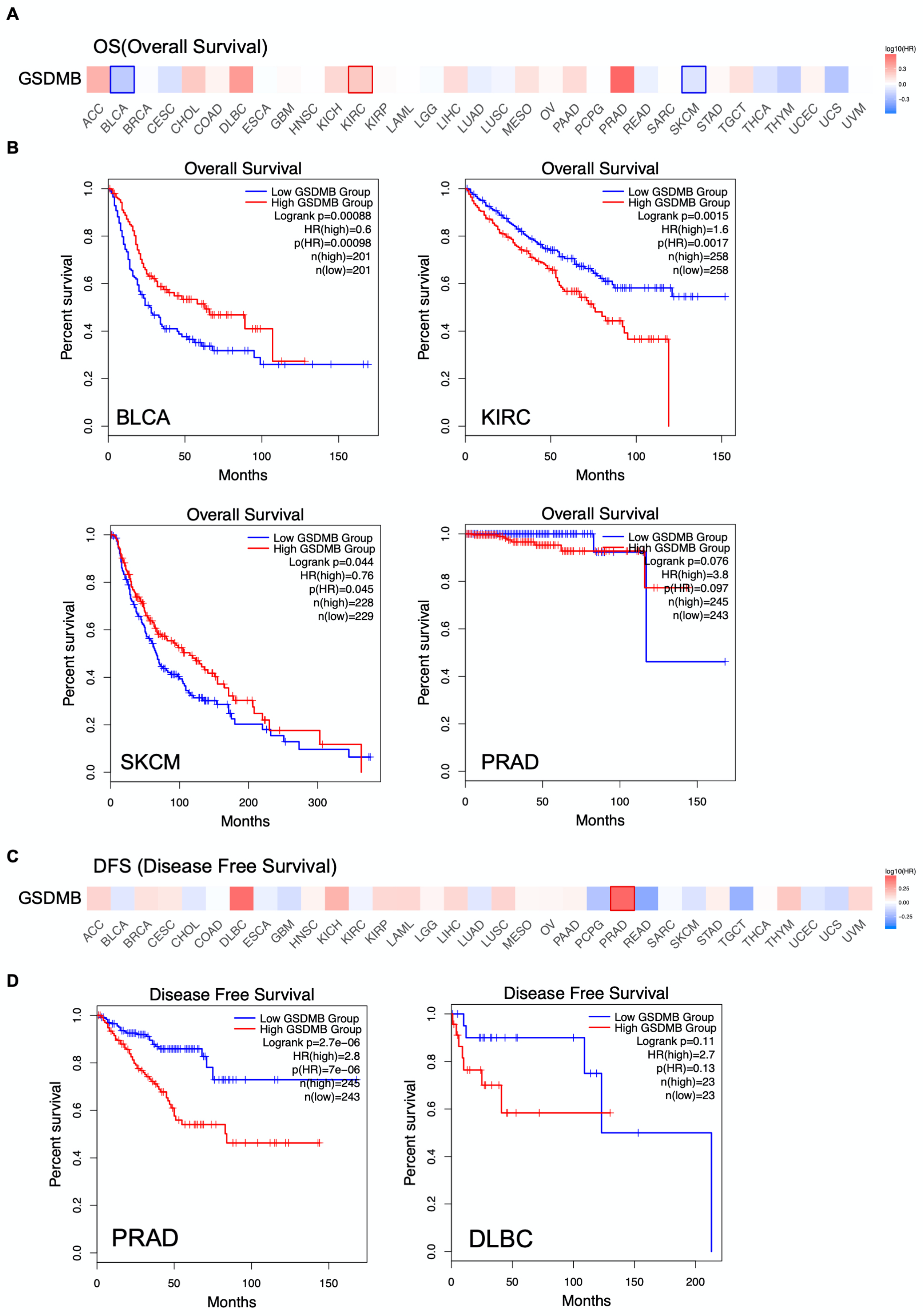

Figure S3. Correlation between mRNA expression of GSDMB and survival prognosis of different cancers. The GEPIA2 tool was used to perform Overall Survival (A) and Disease-

Free Survival (C) analyses of different cancers in TCGA. The survival map (B, D) and the Kaplan-Meier curves are shown, respectively. Patients were divided into high and low-expression groups defined by the expression level of the GSDMB gene (median was the cutoff). The high expression of the GSDMB gene in KIRC and PRAD significantly reduced the survival rate of patients, on the contrary, in BLCA and SKCM. And the expression of the GSDMB gene had the most apparent effect on KIRC.

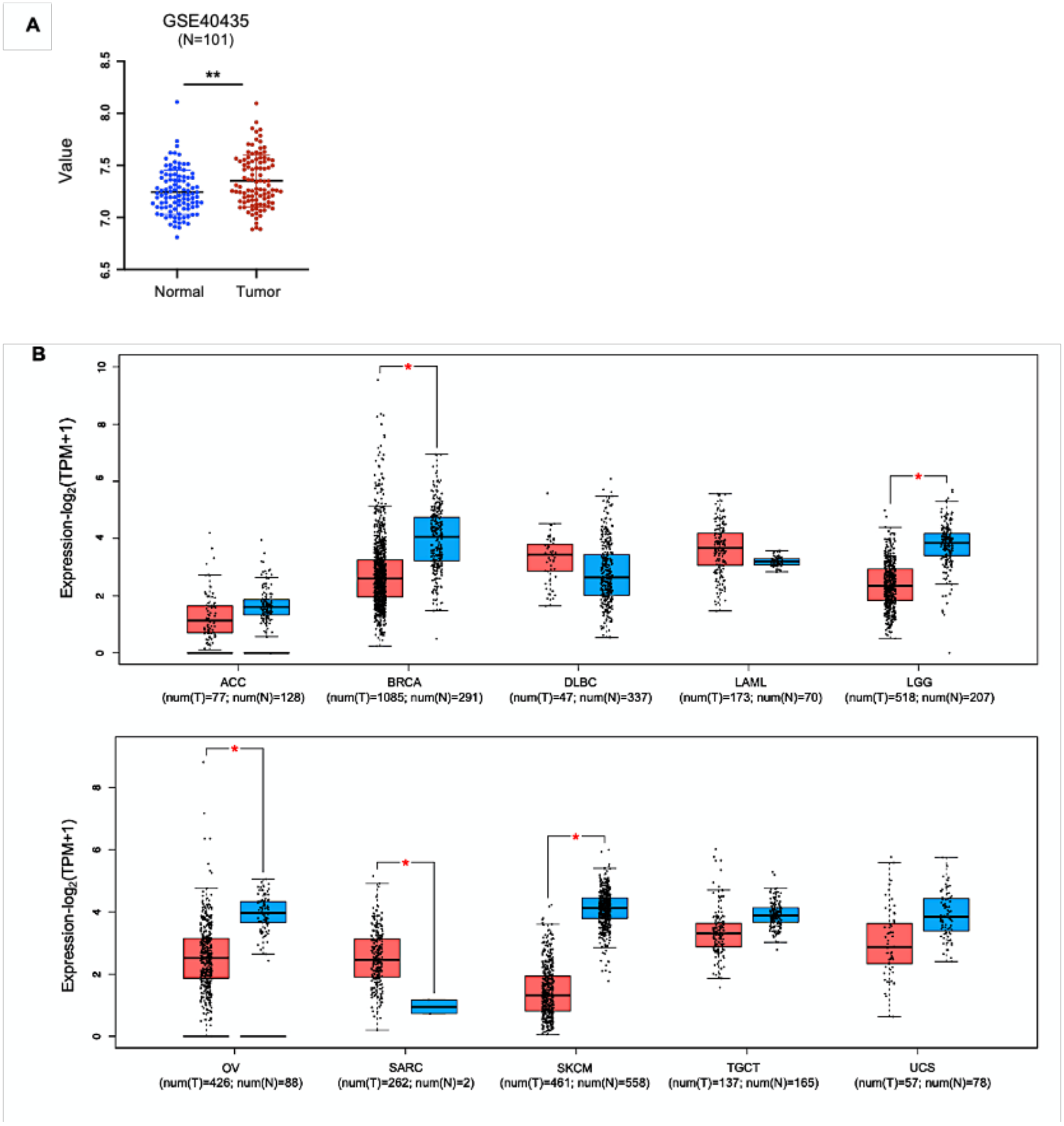

Figure S4. The GSDMB expression level in different tumors. (A) The RNA expression level of GSDMB in ccRCC tumor and its corresponding paracancerous samples were analyzed from the GEO database (GSE40435, n=101). (B) The tumors ACC, BRCA, DLBC, LAML, LGG, OV, SARC, SKCM, TGCT, and UCS in the TCGA and the corresponding normal tissues in the GTEx database were compared, respectively. The box plot data were supplied. $*p < 0.05$; $**p < 0.01$; $***p < 0.001$.

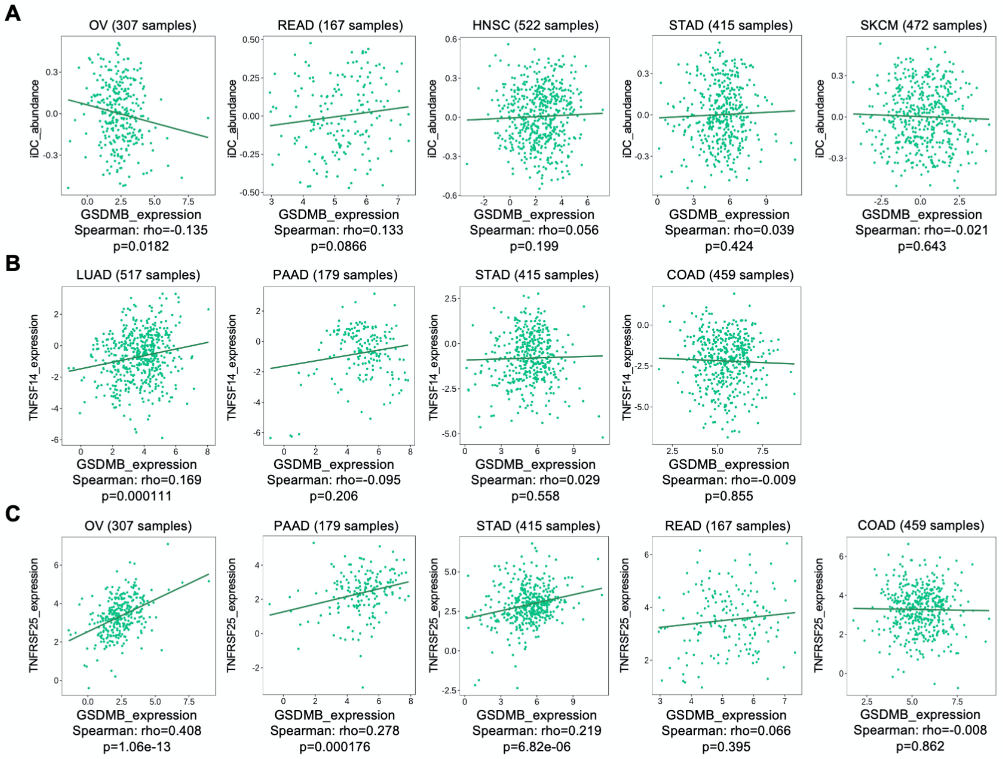

Figure S5. The relationship between GSDMB expression and two kinds of immunomodulators in other tumors. (A) The expression of GSDMB was not related to iDC in OV, READ, HNSC, STAD, and SKCM. (B) The expression of GSDMB was not related to TNFSF14 in LUAD, PAAD, STAD, and COAD. (C) The expression of GSDMB was not related to TNFRSF25 in OV, PAAD, STAD, READ, and COAD.

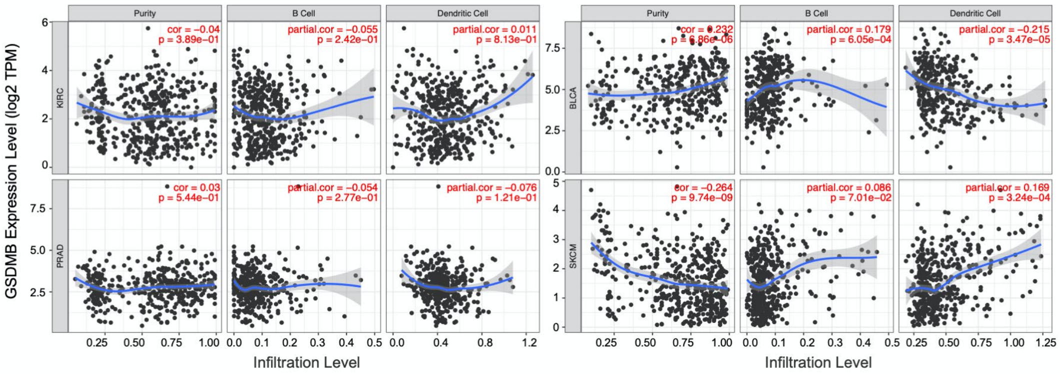

Figure S6. The expression of GSDMB in KIRC, BLCA, PRAD, and SKCM was not correlated with the level of immune infiltration of B cells and dendritic cells. The scatter plots of related cancers generated by Spearman's algorithm within B cell and Dendritic cell in KIRC, BLCA, PRAD, and SKCM.CC
